# Flow-controlled ventilation versus pressure-controlled ventilation in moderate to severe ARDS patients: a randomized crossover physiological study

**DOI:** 10.1186/s40635-025-00847-4

**Published:** 2025-12-24

**Authors:** Julien P. van Oosten, Juliette E. Francovich, Dolf Weller, Wim Rietdijk, Nico Goedendorp, Peter Somhorst, Corstiaan A. den Uil, Diederik Gommers, Annemijn H. Jonkman, Henrik Endeman

**Affiliations:** 1 Intensive Care, Erasmus Medical Center, Intensive Care Volwassenen, Dr. Molewaterplein 40, 3015 GD Rotterdam, The Netherlands; 2 Intensive Care, Maasstad Hospital, Rotterdam, The Netherlands; 3 Department of Hospital Pharmacy, Erasmus Medical Center, Rotterdam, The Netherlands; 4 Intensive Care, OLVG, Amsterdam, The Netherlands

**Keywords:** Flow controlled ventilation, FCV, ICU, Electrical impedance tomography, ARDS

## Abstract

**Background:**

In mechanically ventilated patients with acute respiratory distress syndrome (ARDS) it is of great importance to prevent ventilator-induced lung injury (VILI) using lung protective ventilation. VILI has been associated with a high mechanical power (MP). Flow-controlled ventilation (FCV) could play a role in decreasing the risk of VILI by lowering the MP and preventing atelectrauma by a controlled expiration.

**Objectives:**

To assess the difference in MP between FCV and pressure-controlled ventilation (PCV). Secondary aims were to explore the effect of FCV in terms of ventilation distribution and homogeneity, measured by electrical impedance tomography (EIT).

**Methods:**

Randomized crossover physiological pilot study in ICU patients with a moderate to severe ARDS. Patients were randomized between 90 min of FCV followed by 90 min of PCV, or vice versa. Intratracheal and esophageal pressure, airway flow and EIT were measured continuously, and hemodynamics and venous and arterial blood gases were obtained repeatedly. Pressure–volume loops were constructed for the calculation of the MP.

**Results:**

In 10 patients, optimized FCV (compliance-guided driving pressure) versus PCV resulted in a similar MP (12.6 vs. 14.8 J/min; *p* = 0.302). A stable gas exchange at similar minute volumes was obtained. Optimized FCV resulted in increased tidal ventilation of the mid-ventral to dorsal regions compared to PCV, but EIT demonstrated a trend towards overdistension especially of the non-dependent lung regions. Because of this trend towards overdistension, severe hypercapnia in one patient, and inability to apply FCV as intended, the study was stopped early due to safety concerns.

**Conclusions:**

Optimized FCV compared with PCV resulted in a similar MP and tends towards overdistension in patients with moderate to severe ARDS.

**Trial registration:**

Clinicaltrials.gov identifier: NCT06051188. Registered 22 September 2023.

**Supplementary Information:**

The online version contains supplementary material available at 10.1186/s40635-025-00847-4.

## Background

In mechanically ventilated patients with acute respiratory distress syndrome (ARDS) lung protective ventilation is of uttermost importance to prevent ventilator-induced lung injury (VILI) [1]. Despite current best practice short term mortality in ARDS is still as high as 40% [1]. VILI can be caused by volutrauma (high tidal volumes) and atelectrauma (repeatedly opening and collapsing of alveoli) [2], and has been associated with a high mechanical power (MP), which can partially be modulated by ventilator strategy and settings [3].

Flow-controlled ventilation (FCV) may play a role in decreasing the risk of VILI. In contrast to conventional controlled mechanical ventilation modes with passive expiration, FCV maintains a steady low flow throughout both inspiration and expiration. The gradual (instead of rapid) drop in airway pressure during expiration [2] may prevent alveolar collapse and atelectrauma and therefore could promote lung recruitment, which is especially relevant in ARDS patients with heterogeneous lungs.[4, 5]

In ICU patients with relatively healthy lungs we previously found that FCV compared to pressure-controlled ventilation (PCV) resulted in a lower MP and dissipated energy, more efficient ventilation (lower minute volume with stable PaCO_2_) and a more homogeneous spatial ventilation distribution with increased participation of the dorsal lung regions [6]. By lowering the MP and minute volume and providing an increased ventilation towards the dependent lung regions (suggesting recruitment), FCV could be particularly beneficial for moderate to severe ARDS patients on mandatory ventilation.

Few studies have explored this hypothesis. In porcine (ARDS and non-ARDS) models an increased ventilation efficiency was found [7–9], as well as a reduced alveolar heterogeneity and improved lung aeration on CT-scans [7, 9]. Concerning ARDS patients in the ICU, a lower MP [10] and better oxygenation [11] with FCV were reported as compared to conventional ventilation modes, but methodological concerns exist regarding MP computations in those studies, as we previously noted [6]. Furthermore, the effect of FCV on lung recruitment and homogeneity in ARDS patients have not been investigated.

Therefore, we designed a physiological study comparing FCV and PCV in ventilated patients with moderate to severe ARDS, with the primary aim to assess the difference in MP (hypothesizing a lower MP with FCV). Secondary aims were to explore the differences between FCV and PCV in terms of minute volume, dissipated energy, ventilation distribution and homogeneity, and gas exchange.

## Methods

### Study design and patients

Randomized crossover physiological pilot study at the ICUs of the Maasstad Hospital and Erasmus Medical Center, both located in Rotterdam, The Netherlands, from September 2023 to December 2024 (ClinicalTrials.gov: NCT06051188), approved by the local Medical Ethics Committee (MEC 2023-006). Written informed consent was obtained from the patients’ relatives.

Enrolment criteria were: (1) invasive controlled mechanical ventilation, (2) meeting all Berlin definition criteria for a moderate to severe ARDS [12] and (3) time between ARDS diagnosis and study enrolment ≤ 72 h. Exclusion criteria were: (1) severe sputum stasis, (2) untreated pneumothorax, (3) hemodynamic instability, (4) anticipation of withdrawal of life support, (5) an inner tube diameter ≤ 6 mm, (6) contraindications to electrical impedance tomography (EIT) or an esophageal pressure (Pes) catheter.

### Data collection

At enrollment, we collected sex, age, body mass index (BMI), ideal body weight (IBW), medical history, ARDS etiology, PaO_2_/FiO_2_ ratio, SOFA score, time on non-invasive ventilation or high flow nasal oxygen prior to intubation, time between intubation and study start, and hemodynamic status (norepinephrine dose, central venous oxygen saturation (ScvO_2_) and arterial-venous CO_2_ gap).

### Continuous monitoring

A standard tube adapter (Ventinova Medical BV, The Netherlands) and flow sensor (FluxMed, MBMed, Argentina) were installed between the endotracheal tube and the ventilator tubing. This tube adapter is essential for FCV (Evone ventilator, Ventinova Medical BV) and features a thin pressure probe that extends about 25 cm from the opening of the endotracheal tube, enabling the measurement of intratracheal pressures. By using a connector on the pressure probe (Supplemental Fig. 1) we were able to measure intratracheal pressures while allowing the Evone ventilator to operate. For transpulmonary pressure measurements an esophageal balloon catheter (NutriVent™ Sidam group, Italy) was placed in the mid-esophageal range and inflated aiming for a Baydur occlusion test between 0.9 and 1.1 during calibration. Output of both pressure probes and the flow sensor were connected to a dedicated signal acquisition system (MP160, BIOPAC Systems Inc., USA or FluxMedGrE, MBMmed, Argentina) for synchronized recordings of waveforms sampled at 200 Hz (AcqKnowledge, BIOPAC Systems Inc., USA or FluxView, MBMed, Argentina) during the full protocol. To evaluate the homogeneity of ventilation, continuous monitoring using EIT was commenced with a belt positioned at the 4th-5th intercostal space (Enlight 1800, Timpel Medical, Brazil).

### Study procedures

**Fig. 1:**
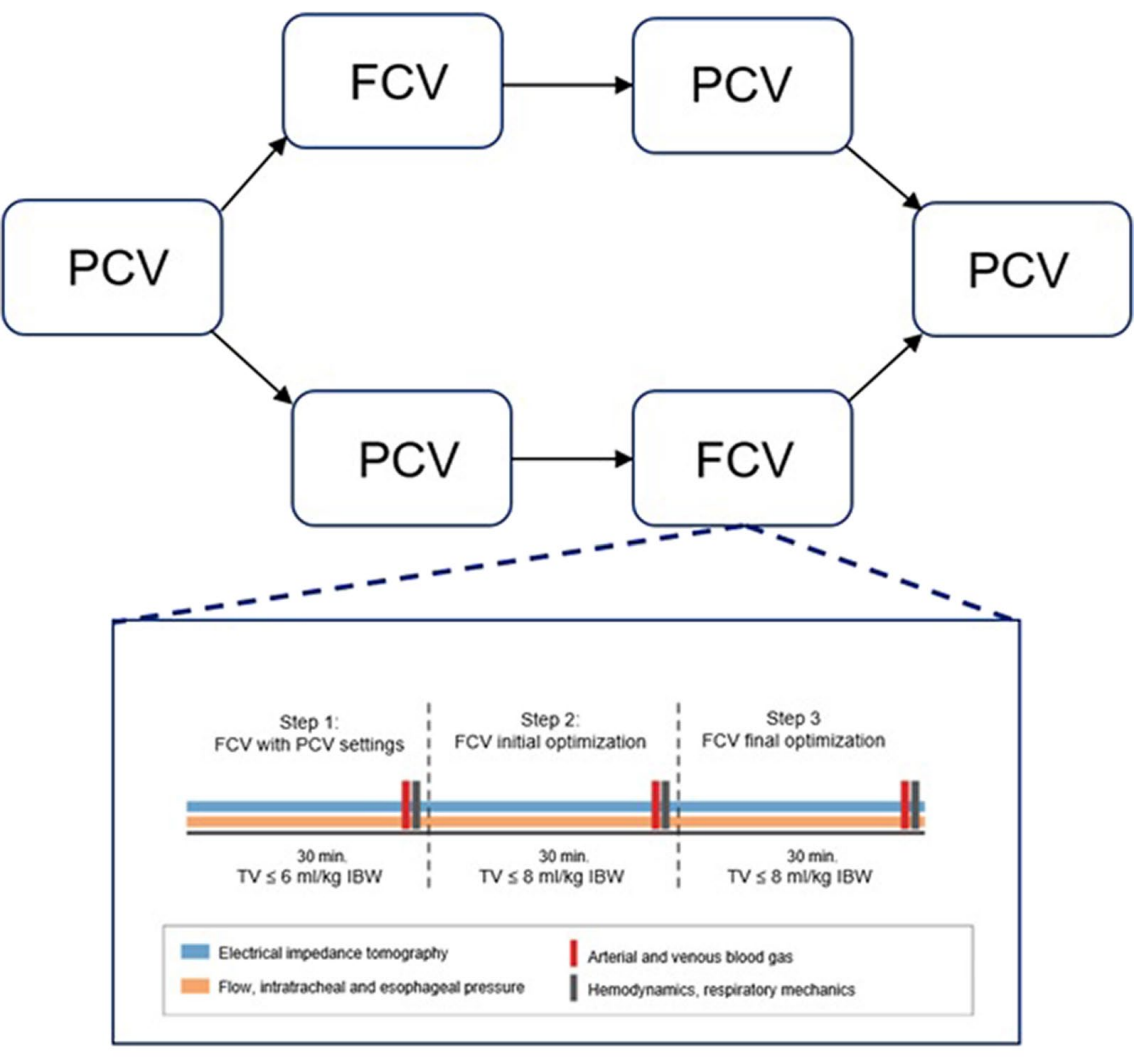
Study procedures with corresponding measurements

The study protocol started within 72 h after intubation and the diagnosis of a moderate to severe ARDS [12]. Patients were randomized between first 90 min on FCV followed by 90 min on PCV or vice versa, using electronic block randomisation (blocks of 4 and 6). No recruitment maneuvers occurred during the study. Study steps lasted 30 min each (Fig. 1). We obtained arterial blood gases (ABG), static respiratory pressures (via end-inspiratory and end-expiratory holds), central venous blood gases, SpO_2_, and hemodynamic and respiratory mechanics measurements at the end of each step. Target values were: SpO_2_ of 92–96%, PaO_2_ < 15 kPa, end-tidal CO_2_ (EtCO_2_) and PaCO_2_ corresponding to a pH > 7.20. Study steps were as follows (for the FCV – PCV sequence):

- *Baseline: PCV.* Settings were adjusted in accordance with our local protocol: PEEP based on a decremental PEEP trial (with a maximum PEEP of 24 cmH_2_O), targeting the highest dynamic respiratory system compliance, and tidal volumes of ≤ 6mL/kg IBW.
- *Step 1: FCV at ‘similar’ PCV settings. *PCV was changed to FCV maintaining PEEP and FiO_2_ settings. Ppeak was adjusted to achieve equivalent tidal volumes as on PCV. Continuous set flow, which determines minute ventilation, was fine-tuned to ensure stable EtCO_2_ levels. Settings were maintained for a duration of 30 min. Because FCV operates an *I*:*E* ratio of 1:1, the respiratory rate is directly influenced by the combination of the set flow, driving pressure, and the patient's respiratory mechanics.
- *Step 2: FCV initial optimization.* FCV was optimized in accordance with the ABG upon completion of step 1 and the manufacturer's guidelines. PEEP remained constant, while FiO_2_ was adjusted, if needed, based on the PaO_2_ and target values established at baseline. Ppeak was incrementally titrated by 1 cmH_2_O to achieve the highest dynamic compliance: if the tidal volume increased more than anticipated (according to the dynamic compliance) after an increase in Ppeak, Ppeak was further raised by 1 cmH_2_O. This process was repeated until the tidal volumes did not exceed expected levels (resulting in a decrease in dynamic compliance) or until a safety threshold of 8 mL/kg IBW or a transpulmonary driving pressure (∆*P*_L_) of 12 cmH_2_O was attained. Flow was modified to keep PaCO_2_ within the target range. The settings were maintained for 30 min.
- *Step 3: FCV final optimization. *Based on the ABG of step 2, flow and FiO_2_ were adjusted, if necessary, to maintain PaO_2_ and PaCO_2_ within target values.
- *Step 4:* PCV for 90 min, using the baseline settings. Blood gases and pressure measurements were collected every 30 min.

The patient’s management was then resumed as per local clinical protocol. When the patient was randomized to the PCV first group, step 4 was initiated after the baseline measurements. Thereafter steps 1–3 were performed.

### Offline analysis

Computation of parameters was performed for the steps baseline, step 1 and step 3 after completion of the study. For additional details we refer to our previous work on FCV in postoperative ICU patients [6].

#### Flow and pressure tracings

Breath-by-breath analysis of flow, intratracheal pressure and esophageal pressure (Pes) tracings was performed (Python 3.10) for a period of 8–10 min at the end of each step. Transpulmonary pressure (*P*_L_) was computed as intratracheal pressure minus Pes. Inspiratory time (Ti), respiratory rate (RR), tidal volume (the time-integral of inspiratory flow), and minute volume were determined from the flow tracings. Peak pressure (Ppeak), plateau pressure (Pplateau), total positive end-expiratory pressure (total PEEP), airway driving pressure (∆*P*), mean airway pressure (Pmean), end-inspiratory transpulmonary pressure (*P*_Lei_), end-expiratory transpulmonary pressure (*P*_Lee_) and transpulmonary driving pressure (∆P_L_) were derived from the intratracheal pressure and *P*_L_ waveforms during inspiratory and expiratory hold maneuvers. Via static measurements, we calculated the compliance of the respiratory system (*C*_RS_ = tidal volume (ml)/∆*P* (cmH_2_O)), of the lung (*C*_L_ = tidal volume (ml)/∆*P*_L_ (cmH_2_O)) and chest wall (*C*_CW_ = tidal volume (ml)/(∆*P*es (cmH_2_O)). Pressure–volume (PV) loops were constructed using the volume waveform and intratracheal and transpulmonary pressure, to compute the total energy of the respiratory system and lung, respectively. The integral of the PV loops was then multiplied by 0.098 (conversion to Joule (J)) and included the elastic dynamic and resistive components, but not the static part (unknown PEEP volume). The MP (J/min) was calculated by multiplying the total energy per breath by the RR, for both the respiratory system (MP_RS_) and lung (MP_L_). Dissipated energy was computed as the hysteresis area of the PV loops per breath (in J/L).

#### EIT

Pixel-level EIT data was collected and analyzed using custom software created in Python 3.10. A stable period comprising a minimum of 10 breaths was manually identified at the end of each step and for every pixel an average inspiration was calculated. Pixels exhibiting a tidal impedance change (∆*Z*) of at least 15% of the maximum pixel ∆*Z* were included in the analysis, to segment the ventilated lung area and reduce the impact of cardiac-related artifacts [13]. We calculated the difference in end-expiratory lung volume (∆EELV) between PCV and FCV by multiplying the difference in end-expiratory lung impedance (∆EELI) by the volume to impedance ratio (tidal volume/global ∆*Z*) as obtained during PCV [13]. Regions of interest (ROIs; ventral, mid-ventral, mid-dorsal and dorsal) were defined with a physiological approach utilizing the ventilated lung space where, on average, each ROI represented 25% of the total variation in lung impedance [14]. For every step, the global ∆*Z* and regional ∆*Z* (for each ROI) were determined, along with the global and regional static respiratory system compliance (for each ROI) (i.e., ∆*Z*/∆*P*). In addition, we evaluated the overall, spatial, and temporal ventilation homogeneity via the global inhomogeneity (GI) index that computes the heterogeneity of pixel inflations (pixel ∆*Z*) compared to the median [15], regional inspiratory volume distribution [16], and Regional Ventilation Delay Index (RVDi) that compares variability in time of pixels to reach 40% of their maximal ∆*Z* during inflation [17], respectively, as described previously [6].

#### Hemodynamics and gas exchange

Arterial partial oxygen pressure (PaO_2_), arterial partial carbon dioxide pressure (PaCO_2_), arterial partial oxygen pressure/fraction of inspired oxygen (PaO_2_/FiO_2_) ratio, central venous oxygen saturation (ScvO_2_), arterial-venous CO_2_ gap, ventilatory ratio [18] and the dose of norepinephrine were collected at each step.

#### Primary endpoint and secondary exploratory endpoints

Our primary endpoint was the difference in respiratory system MP between PCV and optimized FCV. Secondary endpoints were the difference in EIT parameters EELV, ventilation distribution and homogeneity (including GI and RVDi indexes)), minute volume and ventilatory ratio, dissipated energy, airway and transpulmonary pressures, and gas exchange (including *P*/*F* ratio) and hemodynamics (mean arterial pressure, pulse rate) between PCV and (optimized) FCV.

### Sample size and early stop

With no comparator data, our sample size was based on the difference in MP between PCV and FCV from our study in postoperative ICU patients [6]. We took a mean difference in MP of 1.5 instead of 3.13 (in J/min) (and standard deviation of the difference of 2.91 J/min). Using an online sample size calculation for cross-over studies of the Harvard University [19] (alpha, 0.05; power, 0.80) the sample size was 25 patients. To consider potential early withdrawals for clinical reasons, we aimed to include a total of 28 patients.

Upon recruiting 15 patients of which 10 were eventually enrolled (see “Results”), we prematurely stopped the study because of the inability to apply FCV as intended (step 2 and 3) in most patients. This was noticed at the bedside as a drop in respiratory system compliance upon increasing Ppeak (and hence, inability to optimize FCV as per its working principle) or reaching safety limits for lung protection (max. tidal volumes of 8 mL/kg IBW and ∆*P*_L_ of 12 cmH_2_O). Offline analysis also showed ventral overdistention on EIT (see “Results”), making us to decide for early termination.

### Statistical analysis

Statistical analysis was conducted using SPSS (IBM, Armonk, USA). Continuous variables are presented as mean (standard deviation) or median (interquartile range) depending on the normality of distribution. Normality was assessed with the Shapiro–Wilk test. Categorical variables are presented as number (percentages). Comparisons between steps were made using either repeated measures ANOVA or the related-samples Friedman’s test, based on the distribution of the data. A two-sided *p* value of less than 0.05 was deemed statistically significant.

## Results

### Population and characteristics

In total, 15 patients were recruited for the study, of whom 10 patients were included in the analysis; their main characteristics are presented in Table 1. Five patients were excluded because of reaching a *P*/*F* ratio > 200mmHg between enrolment and start of study (*n* = 3), a major pneumothorax discovered at the start of study making EIT measurements unreliable (*n* = 1) and hemodynamic instability (*n* = 1). The median *P*/*F* ratio at enrolment was 159 mmHg, the median SOFA score was 11 and in total 3 patients were on veno-venous extracorporeal membrane oxygenation (VV-ECMO) during the study, and 1 patient was in prone position.

**Table 1:** Main characteristics of the study population

Characteristic	Total (*N* = 10)
Age, years; median (IQR)	64 (43–72)
Male, sex; *n* (%)	5 (50)
BMI, kg/m^2^; median (IQR)	26.8 (23.4–32.8)
IBW, kg; median (IQR)	70.4 (54.5–82.2)
Medical history; *n* (%)	
Asthma Liver failure Pulmonary metastasis None	1 (10) 1 (10) 1 (10) 7 (70)
ARDS characteristics	
Trigger of ARDS; *n* (%) Bacterial pneumonia Sepsis Surgery (aortic bifurcation graft) Trauma (neurotrauma) Unknown Viral (influenza)	3 (30) 1 (10) 1 (10) 1 (10) 1 (10) 3 (30)
Berlin classification; *n* (%)	
Moderate	6 (60)
Severe	4 (40)
P/F ratio at start study, mmHg; median (IQR)^a^ SOFA score at start study; median (IQR)	159 (110–196) 11 (9–13)
Treatment characteristics	
Days on NIV/HFNO before intubation; median (IQR) Time between intubation and start study, h; median (IQR) Randomization order (FCV-PCV)/(PCV-FCV); *n* (%) Prone position during study; *n* (%) Patient on VV-ECMO during study; *n* (%)	0.5 (0.0–2.3) 27 (23–51) 4 (40)/6 (60) 1 (10) 3 (30)
Hemodynamic status	
Fluid administration during study, Liters; median (IQR) Dose norepinephrine at start study, µg/kg/min; median (IQR)	0.2 (0.1–0.4) 0.09 (0.05–0.46)

ARDS: Acute Respiratory Distress Syndrome; BMI: Body Mass Index; HFNO: High Flow Nasal Oxygen; IBW: Ideal Body Weight (men 50/women 45.5 + (0.91 × (height in cm – 152.4)); IQR: Inter Quartile Range; NIV: non-invasive ventilation; P/F ratio: PaO_2_/FiO_2_ ratio; SOFA score: Sequential Organ Failure Assessment score; VV-ECMO: Veno-venous extracorporeal membrane oxygenation

^a^For the calculation of the P/F ratio at the start of the study the three VV-ECMO patients were excluded

No serious adverse events were reported, however in one patient the study was stopped after 60 min on FCV because of severe hypercapnia resulting in a pH < 7.20 despite FCV optimization (adverse event; see Supplemental file). In this patient we used the measurements at 60 min of FCV as step 3.

### Switch from PCV to FCV with similar settings (PCV vs. FCV step 1)

The results of the comparison between FCV with ‘similar’ PCV settings and PCV can be found in the Supplemental file. FCV with ‘similar’ PCV settings did not affect overall MP_RS_ (12.1 (10.6–14.6) vs. 14.8 (11.1–18.4) J/min, *p* = 0.121) (Supplemental Table 1). However, FCV resulted in a lower MP_L_ (5.2 (4.0–6.4) vs. 6.8 (4.9–8.5) J/min, *p* = 0.008) and dissipated energy of the lung (0.19 (0.08–0.21) vs. 0.25 (0.13–0.29) J/L, *p* = 0.003) (values for FCV and PCV, respectively) (Supplemental Table 1). The EELV increased by 16.1 ml (1.6–25.2, *p* = 0.007) during FCV compared to PCV. Although the GI index did not change (Supplemental Table 2), FCV with similar PCV settings showed a trend towards a more homogeneous spatial ventilation distribution with increased participation of the dorsal lung regions using EIT (Supplemental Fig. 2).

### FCV optimization (PCV vs. FCV step 3)

FCV settings were optimized to utilize the potential benefit of this mode (i.e., tidal recruitment followed by controlled expiration to keep the lungs open). When compared with PCV, compliance-optimized FCV application resulted in utilization of higher airway ∆P (14.3 (12.4–14.8) vs. 10.4 (8.6–15.0) cmH_2_O, *p* = 0.007) and higher ∆P_L_ (9.8 (9.1–12.4) vs. 7.6 (6.2–11.7) cmH_2_O, *p* = 0.014), values for FCV vs. PCV, respectively. This resulted in higher tidal volumes on FCV compared to PCV (6.3 (3.7–7.1) vs. 4.9 (4.1–5.6) ml/kg IBW, *p* = 0.059) at a lower respiratory rate (16.7 (12.0–24.2) vs. 23.0 (15.2–30.0) per minute, *p* = 0.034) but similar minute volume (6.6 (5.3–8.6) vs. 7.6 (5.1–9.7) L/min, *p* = 0.125) while oxygenation (PaO_2_ and PaO_2_/FiO_2_), PaCO_2_ and hemodynamics remained stable (Table 2). Optimization of FCV did not affect the overall MP_RS_ (12.6 (10.8–15.3) vs. 14.8 (11.1–18.4) J/min, *p* = 0.302) but did result in a lower MP_L_ compared to PCV (5.4 (4.4–8.3) vs. 6.8 (4.9–8.5) J/min, *p* = 0.034), for FCV vs. PCV, respectively. Optimized FCV did not result in a lower dissipated energy of the lung when compared with PCV (0.20 (0.10–0.32) vs. 0.25 (0.13–0.29) J/L, *p* = 0.795).

**Table 2:** Results PCV vs. optimized FCV

	PCV median (IQR)	FCV step 3 median (IQR)	P-value
*Respiratory parameters*			
Inspiratory TV/IBW (mL)	4.9 (4.1–5.6)	6.3 (3.7–7.1)	0.059
Expiratory TV/IBW (mL)	4.9 (4.2–5.6)	6.3 (3.8–7.1)	0.064
Δ*P* (cmH_2_O)	10.4 (8.6–15.0)	14.3 (12.4–14.8)	0.007
PEEP set (cmH_2_O)	13.0 (9.5–16.5)	13.0 (9.5–16.5)	1.000
PEEP total (cmH_2_O)	14.7 (11.2–16.9)	14.9 (11.3–16.7)	0.390
Ppeak set (cmH_2_O)	27.0 (24.0–32.0)	28.0 (25.5–30.3)	0.853
Ppeak measured (cmH_2_O)	25.7 (23.8–31.7)	29.3 (26.9–31.7)	0.025
Pplateau (cmH_2_O)	24.3 (23.3–29.9)	28.2 (25.0–30.9)	0.010
Pmean (cmH_2_O)	18.5 (15.9–22.9)	21.3 (17.3–23.7)	0.012
*I*:*E* ratio	0.65 (0.57–0.81)	0.89 (0.84–0.98)	0.004
*C*_static_ RS (ml/cmH_2_O)	32.7 (20.7–41.2)	28.4 (18.4–44.4)	0.129
Resistance (cmH_2_O/L/s)^a^	14.8 (12.6–19.8)	8.9 (7.0–10.1)	0.089
RR (x/min)	23.0 (15.2–30.0)	16.7 (12.0–24.2)	0.034
MV (L/min)	7.6 (5.1–9.7)	6.6 (5.3–8.6)	0.125
End inspiratory P_L_ (cmH_2_O)	12.0 (9.7–13.9)	13.7 (12.2–14.6)	0.024
End expiratory P_L_ (cmH_2_O)	4.0 (1.9–5.1)	3.0 (2.4–5.0)	0.688
Δ*P*_L_ (cmH_2_O)	7.6 (6.2–11.7)	9.8 (9.1–12.4)	0.014
C_static_ lung (ml/cmH_2_O)	43.4 (27.1–60.5)	47.1 (24.2–58.9)	0.659
C_static_ chest wall (ml/cmH_2_O)	162 (136–234)	143 (96–190)	0.617
MP_RS_ (J/min)^c^	14.8 (11.1–18.4)	12.6 (10.8–15.3)	0.302
MP_L_ (J/min)	6.8 (4.9–8.5)	5.4 (4.4–8.3)	0.034
Dissipated energy RS (J/L)	0.28 (0.13–0.40)	0.25 (0.16–0.37)	0.909
Dissipated energy lung (J/L)	0.25 (0.13–0.29)	0.20 (0.10–0.32)	0.795
*Gas exchange parameters*			
P/F ratio^b^ (mmHg)	149 (131–194)	155 (128–200)	0.982
PaO_2_ (kPa)	11.6 (10.9–13.0)	12.0 (10.7–13.7)	0.292
PaCO_2_ (kPa)	6.1 (5.6–6.4)	6.1 (5.6–7.4)	0.066
Ventilatory ratio	1.4 (0.8–1.9)	1.1 (0.9–1.7)	0.375
*Hemodynamic parameters*			
Arterial-venous delta CO_2_ (kPa)	0.64 (− 0.21–0.73)	0.54 (0.42–0.65)	0.803
ScvO_2_ (%)	81.4 (79.9–86.2)	83.1 (80.5–86.6)	0.622
Pulse rate (beats/min)	69 (59–99)	79 (62–93)	1.000
Mean arterial pressure (mmHg)	79.0 (73.8–91.3)	82.0 (73.5–86.8)	1.000
Dose norepinephrine (µg/kg/min)	0.10 (0.06–0.38)	0.10 (0.05–0.40)	0.560

ΔP: airway driving pressure; ΔP_L_: transpulmonary driving pressure; Cstatic RS: Static Compliance Respiratory System; Cstatic lung: Static Compliance of the Lung; FCV: Flow-Controlled Ventilation; IBW: Ideal Body Weight (men 50/women 45.5 + (0.91 × (height in cm – 152.4)); I:E ratio: inspiratory:expiratory ratio; IQR: Inter Quartile Range; MV: Minute Volume; MP_L_: Mechanical Power of the lung; MP_RS_: Mechanical Power of the respiratory system; PCV: Pressure-Controlled Ventilation; PEEP: Positive End Expiratory Pressure; P/F ratio: PaO_2_/FiO_2_ ratio; P_L_: Transpulmonary Pressure; RS: Respiratory System; ScvO2: Central Venous Oxygen Saturation; TV: Tidal Volume

^a^Resistance as measured by the Drager (PCV) and Evone (FCV) ventilators

^b^For the calculation of the P/F ratio the three VV-ECMO patients were excluded

^c^Primary endpoint of study

**Fig. 2:**
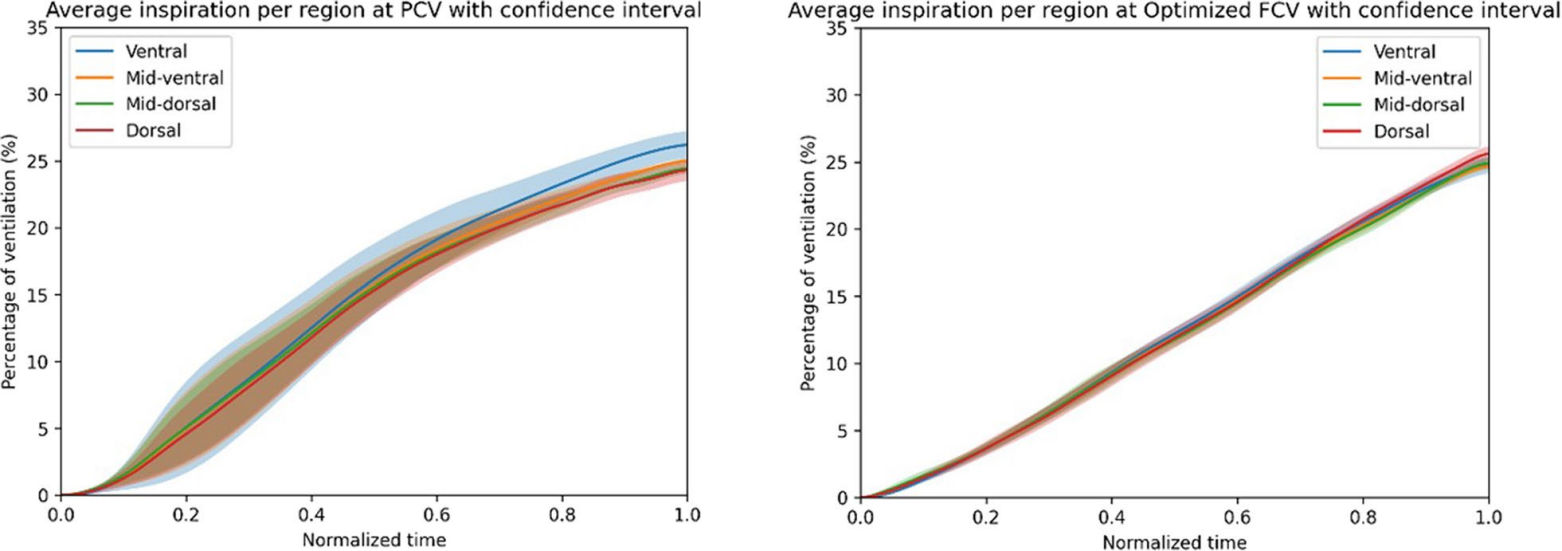
Continuous regional volume distribution: average normalized impedance waveforms with 95% confidence interval per ROI over time and as a percentage of the global ∆*Z*. **A** During PCV, **B** During optimized FCV (step 3). Compared to PCV at baseline, optimized FCV resulted in a more homogeneous spatial ventilation distribution with increased participation of the dorsal lung regions

**Fig. 3:**
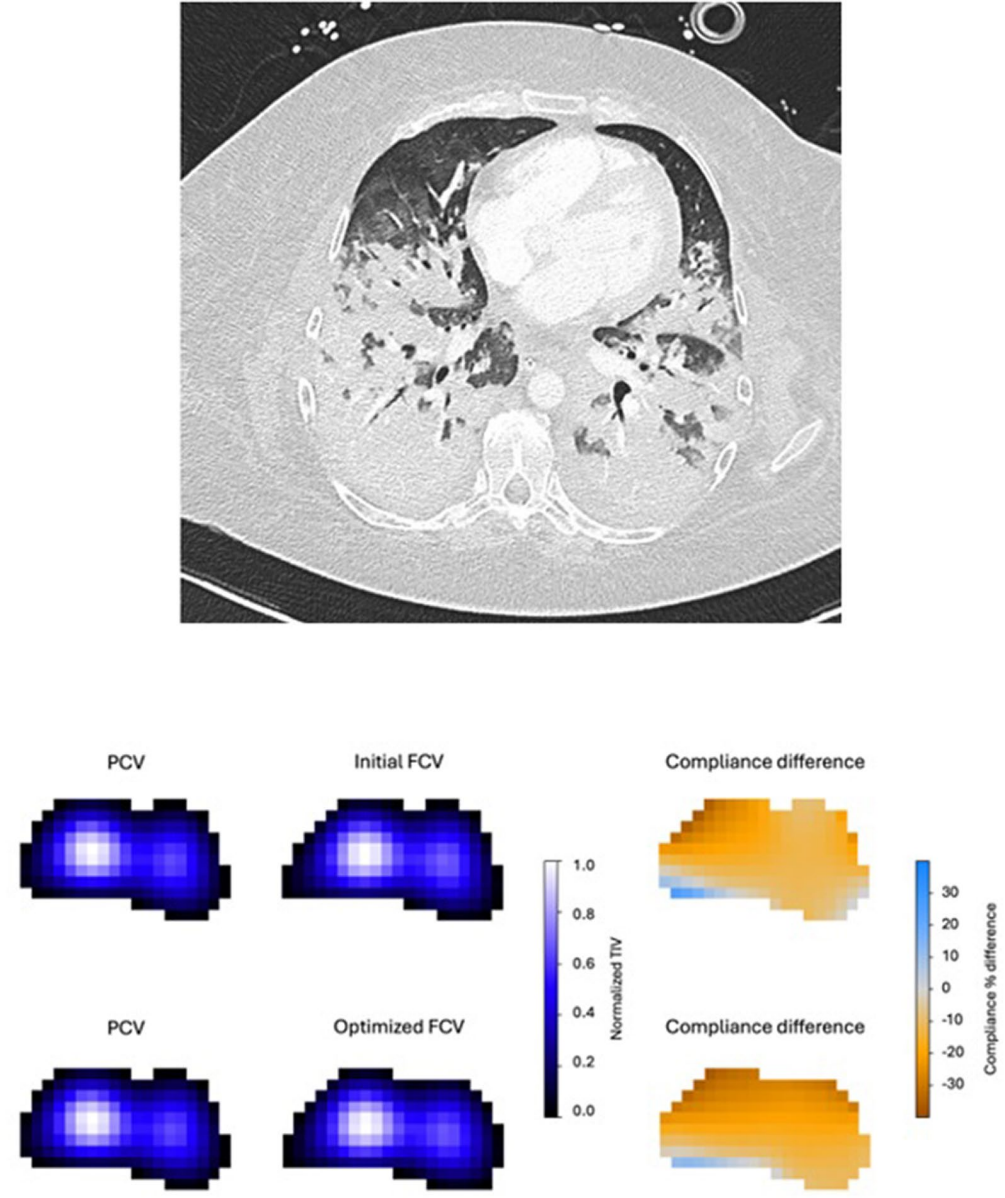
Example of a patient on PCV and optimized FCV. Although optimized FCV resulted in a small increase in tidal volume expressed by the increase in normalized tidal impedance variation (TIV; left), overdistension was detected in all lung regions expressed by the decrease in pixel compliance (right)

EIT results are presented in Table 3. The EELV increased by 21.4 (4.7–27.9) ml (*p* = 0.003) during FCV compared to PCV. Overall homogeneity expressed by the GI index did not change, but optimized FCV compared to PCV did result in an increase in the contribution of the dorsal/dependent lung areas to tidal ventilation (Table 3 and Fig. 2). However, this was accompanied by a trend towards a decrease in compliance in 8 patients of particularly the ventral/nondependent lung parts, suggesting overdistension (Table 3 and Fig. 3). In the other 2 patients the compliance did not change.

**Table 3:** EIT results PCV vs optimized FCV; values represent median (IQR)

a Changes in EIT parameters during FCV as compared to PCV^a^		
	Optimized FCV	P-value
Change in EELV (ml)	21.4 (4.7–27.9)	0.003
Global change in Δ*Z* (%)	30.4 (− 12.6 to 44.3)	
Regional change in Δ*Z* (%)		
ROI ventral ROI mid-ventral ROI mid-dorsal ROI dorsal	14.1 (− 10.0 to 45.8) 26.4 (− 12.7 to 40.1) 33.1 (− 13.0 to 42.0) 34.1 (− 9.2 to 66.7)	0.072 0.038 0.030 0.020
Global change in static compliance (%)	− 9.8 (− 13.9 to 0.4)	
Regional change in static compliance (%)		
ROI ventral ROI mid-ventral ROI mid-dorsal ROI dorsal	− 15.6 (− 26.3 to − 2.8) − 12.5 (− 18.4 to 0.0) − 11.2 (− 15.6 to − 1.5) − 3.1 (− 15.2 to 9.5)	0.060 0.085 0.151 0.451

b Absolute EIT parameters reflecting lung and ventilation homogeneity			
	PCV	Optimized FCV	P-value
GI (%)	40.6 (39.9–49.5)	41.3 (39.9–47.7)	0.671
RVDi (%)	1.70 (1.20–4.32)	3.15 (2.48–4.43)	0.109

a.u.: arbitrary units; ΔZ: tidal impedance change; EIT: Electrical Impedance Tomography; EELV: end-expiratory lung volume; FCV: Flow-Controlled Ventilation; GI: Global Inhomogeneity index; PCV: Pressure-Controlled Ventilation; ROI: Region Of Interest; RVDi: Regional Ventilation Delay index

^a^Changes in ΔZ and static compliance are expressed as percentage change between FCV step 3 and PCV, since both are expressed in arbitrary units which makes direct comparisons between patients unreliable

## Discussion

This study found that optimized FCV compared to PCV in patients with moderate to severe ARDS provides ventilation with a lower MP of the lung, but not of the respiratory system (MP_RS_; primary endpoint). As exploratory secondary endpoints a stable gas exchange can be achieved with FCV at a similar minute volume and with lower dissipated energy (PCV vs. FCV step 1). FCV did not provide a better overall lung homogeneity, however, resulted in a more homogeneous spatial ventilation distribution. Last, optimizing FCV (step 3) resulted in the use of larger tidal volumes and (transpulmonary) pressures. EIT revealed that these larger tidal volumes were mainly distributed to the dorsal lung regions during tidal inflation, and that mostly the ventral/non-dependent lung areas suffered from overdistension.

MP is linked to clinical outcomes in patients with and without lung injury [1, 20]. Our results align with Grassetto et al. [10] and Spraider et al. [21] who also demonstrated a significantly lower MP on FCV compared to conventional ventilation modes in ARDS, but differences exist. They measured pressures and volumes before the tube with VCV or PCV, and after the tube during FCV [10, 21], making comparisons unreliable due to tube resistance. Like our previous work [6], we obtained pressure and flow data at the same location for FCV and PCV, allowing for accurate respiratory mechanics comparison and detailed PV loops for MP and energy dissipation, unlike previous studies relying on bedside MP formulas [10, 21]. We also measured transpulmonary pressures, to separate MP and energy calculations for the lungs and chest wall. When compared with our prior study [6], we did not find a lower MP and dissipated energy of the respiratory system with optimized FCV as compared to PCV. Instead, only the MP of the lung was lower during FCV, possibly due to a difference in compliance of the lung between both patient groups. The lower lung compliance in the ARDS patients compared to postoperative ICU patients made compliance-driven tidal volume optimization during FCV only possible to a limited extent. This can at least partly explain the difference in minute volume and MP between the ARDS and postoperative ICU patient groups.

FCV led to more uniform spatial ventilation, enhancing dorsal lung region participation during tidal inflation, although overall ventilation homogeneity remained unchanged. However, FCV required larger tidal volumes and pressures and did not result in a clinical relevant amount of dorsal recruitment, since oxygenation and dorsal compliance measured by EIT did not change. However, FCV was hindered by overdistension of the ventral/non-dependent lung areas, demonstrated by a decrease in regional compliance on EIT. One of the explanations for the trend towards ventral overdistension could be the absence in relevant recruitment during FCV (i.e., EELV increased only 21 ml during optimized FCV). Besides, by a controlled expiration FCV results in a higher mean airway pressure as compared to PCV, leading to overdistension ventrally, especially during high tidal volumes when the upper inflection point is exceeded. This could also be the reason why we had to stop FCV after 60 min in one patient because of severe hypercapnia leading to a pH < 7.20 (adverse event).

Concerning lung recruitment and homogeneity of ventilation on FCV in ARDS patients so far only studies were performed in porcine ARDS models [7, 8]. Schmidt et al. [7] found that 3 h of FCV significantly reduced nonaerated lung tissue without overinflating other regions. Conversely, Abram et al. [8] randomized pigs to PCV or FCV for 2 h and found no significant differences in lung tissue aeration. Differences in findings may stem from using EIT instead of CT for measurements, and variations in study timing and pathophysiology.

The CT-scan uses Hounsfield units (HU) to assess lung aeration, while we measured lung regional compliance by dividing tidal impedance variation by driving pressure, likely yielding different results. Overdistension on a CT-scan is defined as HU values between—900 and -1000 in the study of Schmidt et al. [7], where a decrease in HU value of the pixels between these HU-values is always indicated as overdistension. In contrast, EIT will show a decrease in compliance between these HU-values as progressive overdistension [13]. Increased driving pressure and tidal volume during FCV was reasoned to lead to overdistension in high-compliance regions, which was further strengthened by comparing EIT compliance maps with CT-scans (Fig. 3). This trend towards overdistension of the ventral lung areas was also evident with FCV at similar tidal volumes to PCV, possibly explaining the lack of effect of FCV on ventilation efficiency.

The difference in lung recruitment found in the animal studies and our ICU trial can be explained by the timing of the study measurements. In pigs, ARDS was induced with oleic acid and directly thereafter study measurements were performed [7, 8], while in our study measurements were performed after a median of 27 h after intubation. This difference in duration of ARDS is expected to influence recruitability, where ARDS lungs may be better recruitable even earlier in their disease state (exudative vs. consolidative phase of ARDS) [22]. Finally, the porcine oleic acid model results in a secondary and rather homogenous ARDS, while our ARDS patients suffer mostly from a primary and heterogenous ARDS.

This is the first physiological study in patients with a moderate to severe ARDS that evaluates the differences in MP, dissipated energy, and detailed ventilation distribution between FCV and PCV via advanced respiratory monitoring. The randomized pilot crossover study design helps to rule out the effect of time on lung recruitment and thereby ventilatory (in)efficiency. However, our study does have some limitations. First, we did not reach our full sample size and therefore results should be interpreted with caution. Since we introduced limits for lung-protective ventilation to prevent any potential harm/VILI to this vulnerable patient category, we decided to stop the study early when we noticed ventral overdistension in most patients. Second, compared to Spraider et al. [9] who found a lower PEEP level with FCV compared to PCV (3 vs. 5 cmH_2_O) in their porcine model, we choose not to perform a compliance-guided PEEP titration on FCV. PEEP was optimized at baseline PCV as per our standard practice. By performing another compliance-guided PEEP titration on FCV, EIT parameters including our secondary endpoint EELV as a measurement of recruitment/derecruitment could not be interpreted between modes, because the EELV is directly influenced by the set PEEP level. Furthermore, in our previous study on FCV in postoperative ICU patients [6] we did not measure a difference in total PEEP during an expiratory hold maneuver between PCV and FCV (8.3 and 8.4 cmH_2_O, respectively) when using the same set PEEP level, and therefore did not expect intrinsic PEEP to be different between ventilation modes in the current study. Indeed, also in ARDS patients we did not measure a difference in total PEEP (at the same set PEEP) between PCV and (optimized) FCV, as measured during an expiratory hold maneuver. Third, we performed FCV only for 90 min which could be too short for slow recruitment to take place. However, for other parameters like the MP, minute volume and ventilation homogeneity the difference between PCV and FCV became clear early in the study. The total study duration of 3 h was also chosen for practical reasons, e.g. maximum recording of 180 min for EIT machine without restart/recalibrations (that could affect EIT results).

In conclusion, optimized FCV, compared to PCV, in patients with moderate to severe ARDS resulted in a significant lower MP of the lung, but not of the respiratory system. Although FCV resulted in a more homogeneous spatial ventilation distribution with increased participation of the dorsal lung regions during inflation with larger tidal volumes, FCV tended towards overdistension of the non-dependent lung areas. This trend towards overdistension together with the inability to apply FCV as intended (resulting in severe hypercapnia and early stop in one patient) made us decide to terminate the study preterminal. These findings are contrasting the general hypothesis that FCV could promote lung recruitment and raise concerns about the suitability of FCV for the management of ARDS patients in the ICU.

## Supplementary Information

Supplementary material 1.

## Data Availability

The datasets used and/or analyzed during the current study are available from the corresponding author on reasonable request.

## Abbreviations

ABG: Arterial blood gas
ARDS: Acute respiratory distress syndrome
a.u.: Arbitrary units
BMI: Body mass index
COPD: Chronic obstructive pulmonary disease
*C*_CW_: Compliance of the chest wall
*C*_L_: Compliance of the lung
*C*_RS_: Compliance respiratory system
∆*P*: Airway driving pressure
∆*P*_L_: Transpulmonary driving pressure
∆*Z*: Tidal impedance change (∆*Z*)
EELI: End-expiratory lung impedance
EELV: End-expiratory lung volume
EIT: Electrical impedance tomography
EtCO_2_: End-tidal CO_2_
FCV: Flow-controlled ventilation
FiO_2_: Fraction of inspired oxygen
GI: Global inhomogeneity index
HFNO: High flow nasal oxygen
HU: Hounsfield unit
IBW: Ideal body weight
ICU: Intensive care unit
*I*:*E*-ratio: Inspiratory:expiratory ratio
IQR: Inter quartile range
MP: Mechanical power
MP_L_: Mechanical power of the lung
MP_RS_: Mechanical power of the respiratory system
MV: Minute volume
NIV: Non-invasive ventilation
PaCO_2_: Arterial partial carbon dioxide pressure
PaO_2_: Arterial partial oxygen pressure
PCV: Pressure-controlled ventilation
PEEP: Positive end-expiratory pressure
Pes: Esophageal pressure
*P*/*F* ratio: PaO_2_/FiO_2_ ratio
*P*_L_: Transpulmonary pressure
*P*_Lee_: End-expiratory transpulmonary pressure
*P*_Lei_: End-inspiratory transpulmonary pressure
Pmean: Mean airway pressure
Ppeak: Peak pressure
Pplat: Plateau pressure
PV-loop: Pressure–volume loop
ROI: Region of interest
RR: Respiratory rate
RVDi: Regional ventilation delay index
ScvO_2_: Central venous oxygen saturation
SOFA score: Severe organ failure assessment
SpO_2_: Peripheral oxygen saturation
Ti: Inspiratory time
TV: Tidal volume
VILI: Ventilator-induced lung injury
VV-ECMO: Veno-venous extracorporeal membrane oxygenation
