# Supplementary material for: Flow-controlled ventilation versus pressure-controlled ventilation in moderate to severe ARDS patients: a randomized crossover physiological study
Source: Intensive Care Med Exp. 2025 Dec 24;13:137. doi: 10.1186/s40635-025-00847-4 (PMC12738392; doi:10.1186/s40635-025-00847-4)
Supplement: Supplementary file 1 — Supplementary material 1. [file 40635_2025_847_MOESM1_ESM.docx]

Online Supplement

**Flow-controlled ventilation versus pressure-controlled ventilation in moderate to severe ARDS patients: a randomized crossover physiological study**

Julien P. Van Oosten ^1^, Juliette E. Francovich^1^, Dolf Weller^1,2^, Wim Rietdijk^1,3^, Nico Goedendorp^1^, Peter Somhorst^1^, Corstiaan A. Den Uil^2^, Diederik A.M.P.J. Gommers^1^, Annemijn H. Jonkman^1^, Henrik Endeman^1,4^

**Author affiliations:**

1) Intensive Care, Erasmus Medical Center, Rotterdam, The Netherlands

2) Intensive Care, Maasstad Hospital, Rotterdam, The Netherlands

3) Department of Hospital Pharmacy, Erasmus Medical Center, Rotterdam, The

Netherlands

4) Intensive Care, OLVG, Amsterdam, The Netherlands

EXTENDED RESULTS SECTION

**Population and characteristics**

In total, 15 patients were recruited for the study, of whom 10 patients were included in the analysis; their main characteristics are presented in **Table 1.** Five patients were excluded because of reaching a P/F ratio > 200mmHg between enrolment and start of study (n=3), a major pneumothorax discovered at the start of study making EIT measurements unreliable (n=1) and hemodynamic instability (n=1). The median P/F ratio at enrolment was 159 mmHg, the median SOFA score was 11 and in total 3 patients were on veno-venous extracorporeal membrane oxygenation (VV-ECMO) during the study, and 1 patient was in prone position.

No serious adverse events were reported, however in one patient the study was stopped after 60 minutes on FCV because of severe hypercapnia resulting in a pH < 7.20 despite FCV optimization (adverse event). The patient with an ARDS of unknown origin was stable for 90 minutes on PCV (PEEP 10 cmH_2_O, Ppeak 24cmH_2_O, RR 30min^-1^), resulting in a tidal volume of 252 ml and PaCO_2_ of 6.71 kPa. During step 1 FCV was initiated with the same PEEP and Ppeak settings, but this resulted in a PaCO_2_ of 7.26 kPa (flow 20L/min, RR 28 min^-1^). During step 2 we were not able to optimize FCV as intended, because compliance decreased while increasing the driving pressure. Despite a Ppeak of 27cmH_2_O, maximum flow of 20L/min (resulting in a tidal volume of 228ml, RR of 28 min^-1^) the PaCO_2_ increased to 7.94. To prevent further respiratory acidosis FCV was stopped. In this patient we used the measurements at 60 minutes of FCV as step 3.

**Switch from PCV to FCV with similar settings (PCV vs. FCV step 1)**

FCV with ‘similar’ PCV settings did not affect overall MP_RS_ (12.1 (10.6-14.6) vs. 14.8 (11.1-18.4) J/min, p=0.121) and dissipated energy (0.23 (0.14-0.27) vs. 0.28 (0.13-0.40) J/L, p=0.363). However, FCV resulted in a lower MP_L_ (5.2 (4.0-6.4) vs. 6.8 (4.9-8.5) J/min, p=0.008) and dissipated energy (0.19 (0.08-0.21) vs. 0.25 (0.13-0.29) J/L, p=0.003) of the lung (values for FCV and PCV, respectively). For all results comparing FCV step 1 and PCV, see **Supplemental Tables 1 and 2.**

The EELV increased by 16.1 ml (1.6-25.2, p=0.007) during FCV compared to PCV (**Supplemental Table 2**). Although the GI index did not change (**Supplemental Table 2**), FCV with similar PCV settings showed a trend towards a more homogeneous spatial ventilation distribution with increased participation of the dorsal lung regions using EIT (**Supplemental Figure 2**). However, this was accompanied by a decrease in compliance of most regions of interest in 8 patients, particularly in the ventral/non-dependent lung parts, suggesting overdistension (**Supplemental Table 2**). In the other 2 patients the compliance did not change or slightly increased.

**Supplemental Table 1.** Results PCV vs. FCV with PCV settings (step 1)

|  | **PCV**  **Median (IQR)** | **FCV step 1**  **Median (IQR)** | **P-value** |  |
| --- | --- | --- | --- | --- |
| ***Respiratory parameters*** | | | |  |
| Inspiratory TV/IBW (mL) | 4.9 (4.1-5.6) | 4.8 (3.6-6.1) | 0.783 |  |
| Expiratory TV/IBW (mL) | 4.9 (4.2-5.6) | 4.7 (3.7-6.1) | 0.869 |  |
| ΔP (cmH_2_O) | 10.4 (8.6-15.0) | 11.6 (9.2-14.6) | 0.502 |  |
| PEEP set (cmH_2_O) | 13.0 (9.5-16.5) | 13.0 (9.5-16.5) | 1.000 |  |
| PEEP total (cmH_2_O) | 14.7 (11.2-16.9) | 14.9 (12.1-16.8) | 0.388 |  |
| Ppeak set (cmH_2_O) | 27.0 (24.0-32.0) | 25.5 (24.0-30.3) | 0.128 |  |
| Ppeak measured (cmH_2_O) | 25.7 (23.8-31.7) | 26.5 (25.9-31.7) | 0.053 |  |
| Pplateau (cmH_2_O) | 24.3 (23.3-29.9) | 24.3 (23.7-30.7) | 0.194 |  |
| Pmean (cmH_2_O) | 18.5 (15.9-22.9) | 19.2 (17.6-23.7) | 0.052 |  |
| I:E ratio | 0.65 (0.57-0.81) | 0.94 (0.84-0.96) | 0.002 |  |
| C_static_ RS (ml/cmH_2_O) | 32.7 (20.7-41.2) | 30.2 (18.1-41.7) | 0.441 |  |
| Resistance (cmH_2_O/L/s)^#^ | 14.8 (12.6-19.8) | 8.6 (7.2-10.6) | 0.007 |  |
| RR (x/min) | 23.0 (15.2-30.0) | 18.9 (14.3-25.7) | 0.122 |  |
| MV (L/min) | 7.6 (5.1-9.7) | 6.3 (5.2-8.4) | 0.117 |  |
| End inspiratory P_L_ (cmH_2_O) | 12.0 (9.7-13.9) | 11.5 (10.4-14.0) | 0.485 |  |
| End expiratory P_L_ (cmH_2_O) | 4.0 (1.9-5.1) | 3.3 (1.9-4.5) | 0.471 |  |
| ΔP_L_ (cmH_2_O) | 7.6 (6.2-11.7) | 8.6 (6.4-13.2) | 0.965 |  |
| C_static_ lung (ml/cmH_2_O) | 43.4 (27.1-58.1) | 42.6 (26.5-59.1) | 0.685 |  |
| C_static_ chest wall (ml/cmH_2_O) | 162 (136-234) | 163 (144-229) | 0.803 |  |
| MP_RS_ (J/min)^$^ | 14.8 (11.1-18.4) | 12.1 (10.6-14.6) | 0.121 |  |
| MP_L_ (J/min) | 6.8 (4.9-8.5) | 5.2 (4.0-6.4) | 0.034 |  |
| Dissipated energy RS (J/L) | 0.28 (0.13-0.40) | 0.23 (0.14-0.27) | 0.363 |  |
| Dissipated energy lung (J/L) | 0.25 (0.13-0.29) | 0.19 (0.08-0.21) | 0.003 |  |
| ***Gas exchange parameters*** | | | |  |
| P/F ratio* (mmHg) | 149 (131-194) | 150 (124-180) | 0.417 |  |
| PaO_2_ (kPa) | 11.6 (10.9-13.0) | 12.0 (11.5-12.8) | 0.545 |  |
| PaCO_2_ (kPa) | 6.1 (5.6-6.4) | 6.4 (6.1-7.2) | 0.017 |  |
| Ventilatory ratio | 1.4 (0.8-1.9) | 1.3 (0.8-1.6) | 0.365 |  |
| ***Hemodynamic parameters*** | | | |  |
| Arterial-venous delta CO_2_ (kPa) | | 0.64 (-0.21-0.73) | 0.50 (0.40-0.66) | 0.617 |
| ScvO_2_ (%) | | 81.4 (79.9-86.2) | 81.9 (79.6-86.9) | 0.887 |
| Pulserate (beats/min) | | 69 (59-99) | 69 (65-92) | 1.000 |
| Mean arterial pressure (mmHg) | | 79.0 (73.8-91.3) | 76.0 (70.0-84.0) | 0.920 |
| Dose norepinephrine (µg/kg/min) | | 0.10 (0.06-0.38) | 0.10 (0.05-0.42) | 0.363 |

***Abbreviations:*** *ΔP: airway driving pressure; ΔP_L_: transpulmonary driving pressure; Cstatic RS: Static Compliance Respiratory System; Cstatic lung: Static Compliance of the Lung; FCV: Flow-Controlled Ventilation; IBW: Ideal Body Weight (men 50/women 45.5 + (0.91x(height in cm – 152.4)); I:E ratio: inspiratory:expiratory ratio; IQR: Inter Quartile Range; MV: Minute Volume; MP_L_: Mechanical Power of the lung; MP_RS_: Mechanical Power of the respiratory system; PCV: Pressure-Controlled Ventilation; PEEP: Positive End Expiratory Pressure; P/F ratio: PaO_2_/FiO_2_ ratio; P_L_: Transpulmonary Pressure; RS: Respiratory System; ScvO2: Central Venous Oxygen Saturation; TV: Tidal Volume*

*^#^Resistance as measured by the Drager (PCV) and Evone (FCV) ventilators.*

**For the calculation of the P/F ratio the three VV-ECMO patients were excluded.*

*^$^Primary endpoint of study*

**Supplemental Table 2.** EIT results PCV vs. FCV with PCV settings (step 1); values represent median (IQR)

| **Supplemental Table 2a. Changes in EIT parameters during FCV as compared to PCV^*^** | | | |
| --- | --- | --- | --- |
|  | | **FCV step 1** | **P-value** |
| Change in EELV (ml) | | 16.1 (1.6-25.2) | 0.007 |
| Global change in ΔZ (%) | | 0.1 (-19.7-9.5) |  |
| Regional change in ΔZ (%)  ROI ventral  ROI mid-ventral  ROI mid-dorsal  ROI dorsal | | -2.0 (-21.9-5.0)  -1.3 (-18.5-10.2)  0.8 (-12.0-18.6)  5.4 (2.7-34.1) | 0.379  0.999  0.328  0.102 |
| Global change in static compliance (%) | | -10.0 (-13.8-1.1) |  |
| Regional change in static compliance (%)  ROI ventral  ROI mid-ventral  ROI mid-dorsal  ROI dorsal | | -12.0 (-22.5- -3.3)  -12.9 (-18.1-0.5)  -9.2 (-14.0- 9.0)  -2.1 (-11.3-8.3) | 0.070  0.115  0.257  0.424 |
| **Supplemental Table 2b. Absolute EIT parameters reflecting lung and ventilation homogeneity** | | | |
|  | **PCV** | **FCV** | **P-value** |
| GI (%) | 40.6 (39.9-49.5) | 40.4 (39.9-48.4) | 0.603 |
| RVDi (%) | 1.70 (1.20-4.32) | 2.4 (1.7-4.6) | 0.620 |

***Abbreviations:*** *a.u.: arbitrary units; ΔZ: tidal impedance change; EIT: Electrical Impedance Tomography; EELV: end-expiratory lung volume; FCV: Flow-Controlled Ventilation; GI: Global Inhomogeneity index; PCV: Pressure-Controlled Ventilation; ROI: Region Of Interest; RVDi: Regional Ventilation Delay index.*

*^*^Changes in ΔZ and static compliance are expressed as percentage change between FCV step 1 and PCV at baseline, since both are expressed in arbitrary units which makes direct comparisons between patients unreliable.*

**Supplemental Figure 1.**

*
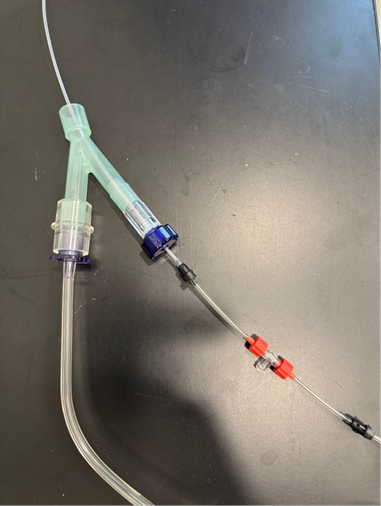
Tube adapter (green) with connector (red) which enables the measurement of intratracheal pressures while allowing the Evone ventilator to operate.*

*
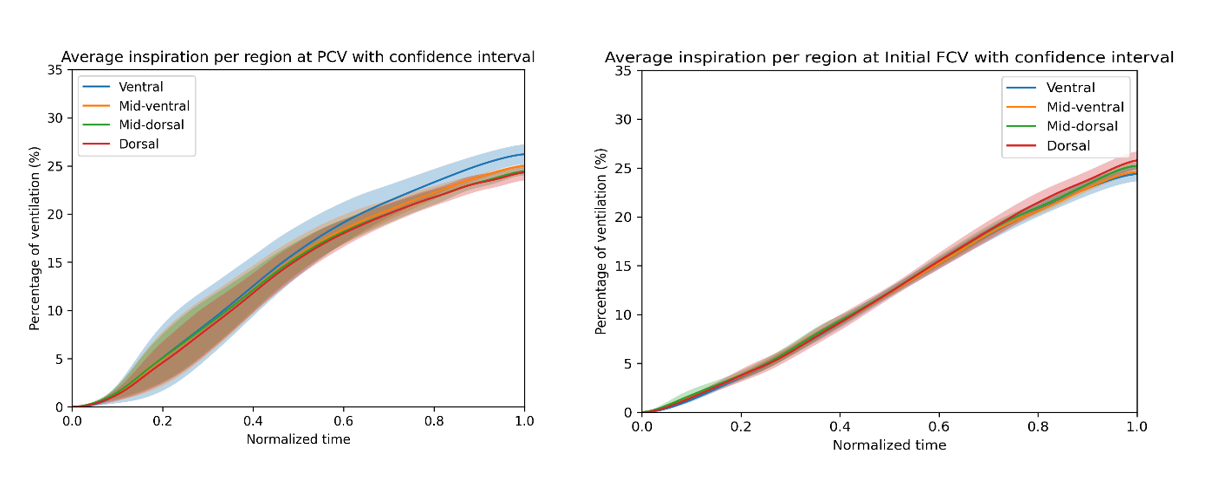
***Supplemental Figure 2.**

*Continuous regional impedance distribution averaged over all patients and per region of interest, in an average inspiration at PCV (left) and step 1 on FCV (right). Shaded areas represent 95% confidence intervals.*
